# Gemcitabine twice weekly as a radiosensitiser for the treatment of brain metastases in patients with carcinoma: a phase I study

**DOI:** 10.1038/sj.bjc.6602444

**Published:** 2005-02-15

**Authors:** A Maraveyas, J Sgouros, S Upadhyay, A-H Abdel-Hamid, M Holmes, M Lind

**Affiliations:** 1 Department of Academic Oncology and University of Hull, Saltshouse Road, Hull HU8 9HE, UK; 2 Department of Clinical Oncology, The Princess Royal Hospital, Hull HU8 9HE, UK

**Keywords:** brain metastases, radiosensitisation, gemcitabine, phase I study

## Abstract

Conventional treatment for brain metastases (BM) is whole-brain radiotherapy (WBRT). Efficacy is poor. It might be increased by a potent radiosensitiser such as gemcitabine which is believed to cross the disrupted blood–brain barrier. Primary objective of this study was to determine the maximum tolerated dose (MTD) of twice weekly gemcitabine given concurrently with WBRT. Patients with BM from carcinoma were included. The dose of WBRT was 30 Gys (10 daily fractions). Gemcitabine was given 2–4 h prior to WBRT on days 1 and 8 for the first cohort of patients and then on days 1, 4, 8 and 11. Starting dose was 25 mg m^−2^, escalated by 12.5 mg m^−2^ increments. At least three patients were included per level. Dose limiting toxicity (DLT) was defined as grade 4 haematological or grade ⩾3 nonhaematological toxicity. A total of 25 patients were included; 74% had a PS 1 (ECOG). In all, 23 had non-small-cell lung cancer, six colorectal, four breast, two renal cell and one oesophageal carcinoma. A total of 92% had concurrent extracranial disease. Six had single BM, 13 had two or three BM and six multiple. Up to 50 mg m^−2^ (level 4) no DLT was observed. At 62.5 mg m^−2^, one out of six patients developed DLT (thrombocytopenia-bleeding). The next dose level (75 mg m^−2^) was abandoned after grade 4 bone marrow toxicity (fatal neutropenic sepsis) was seen in one out of two patients. So that the dose of 50 mg m^−2^ will be taken forward for further study.

Brain metastases (BM) occur in 20–40% of cancer patients during the course of their illness and 50–75% of them have more than one metastasis (Patchel, 2003). Except for very few cases where surgery may be indicated, the mainstream accepted therapeutic modality is whole brain radiotherapy (WBRT). Gamma-knife radiosurgery and conformal radiotherapy are recent developments that may confer a prognosis improvement to selected patients mostly in cases of single BM and controlled extracranial disease. The prognosis of the vast majority of patients who develop BM is poor. The median survival for treated patients with WBRT is approximately 4 months and the 1-year survival only 12% (Lagerwaard *et al*, 1999). Chemotherapy is not usually given and if the patient is actually receiving chemotherapy, it is often discontinued. This attitude has been fostered by the theoretical difficulty of drugs to pass the blood–brain barrier and achieve therapeutic significance in brain micrometastases. This however is probably not the case with macroscopic metastases. Brain metastases in patients with many chemosensitive tumours (e.g. testicular cancer, lymphoma, choriocarcinoma) are well known to respond to chemotherapy. A recent study by Postmus *et al* (2000) in patients with small-cell carcinoma and BM showed a 22% response rate to chemotherapy alone. However, the combination of chemotherapy and radiotherapy produced a 57% response rate and the control in the brain disease in these patients was longer than in patients who received only chemotherapy. A phase III study of early *vs* late WBRT with concurrent cisplatin and vinorelbine in patients with non-small-cell lung cancer (NSCLC) and BM was reported by Robinet *et al* (2001). Patients were chemonaive and drugs were given at full dose. Patients were randomised to receive WBRT 30 Grays (Gy) in 10 fractions over 12 days after two cycles of chemotherapy or on days 1–12 concurrently with chemotherapy. The grade 3–4 toxicities were similar and specifically neurotoxicity and toxic deaths were comparable in both arms. The response rate to chemotherapy (as judged in the patients randomised to delayed WBRT) was 27%. Many other studies have shown that BM from a number of primary sites can be equally chemosensitive as to areas outside the brain (Van den Bent, 2003). The problem of perceived resistance is probably due to the fact that BM usually develop as a late event in patients already treated with many agents to which resistance has already developed.

One of the most promising newer chemotherapeutic drugs is the bifluorinated analogue of cytarabine, gemcitabine. Gemcitabine, both as a single agent and in combination with cisplatin or other agents achieves a significant response rate in NSCLC, head and neck carcinoma, pancreatic, breast and gynaecologic cancer (Carmichael, 1998). This tends to be accompanied by a favourable toxicity profile.

Gemcitabine has also demonstrated activity in BM in patients with carcinoma. Gridelli *et al* (1999) evaluated the efficacy of the drug in 30 patients with NSCLC who had already received chemotherapy with a platinum compound. The authors observed six partial responses, and two of them were in patients with BM.

In a further study by Ceribelli *et al* (2003), again in patients with NSCLC but this time treated with cisplatin and gemcitabine, the overall response rates in the Arm of gemcitabine at the standard 30 min infusion (Arm A) and in the arm of gemcitabine at an infusion rate of 10 mg m^−2^ min^−1^ (Arm B) were 26% (95% confidence interval (95% CI), 10–42%) and 34% (95% CI, 17–52%), respectively. In the patients who happened to have BM, it was interesting to note a high response rate (67%) of BM in Arm B. Toxicity was tolerable and comparable in the two arms. As a radiosensitiser, gemcitabine has been used in patients with NSCLC, pancreatic cancer, head and neck cancer and cervical cancer. There are no data for the use of gemcitabine as a radiosensitiser in the setting of BM.

The aim of the current study was to find the maximum tolerated dose (MTD) of gemcitabine when given as a radiosensitiser on a twice weekly schedule to patients with BM from carcinoma.

## MATERIALS AND METHODS

### Eligibility

Patients with histologically or cytologically confirmed carcinoma and BM were eligible for the study provided that surgery was not an option. Eligibility criteria also included an Eastern Cooperative Oncology Group performance status of 0–2 and age ⩾18 years. Adequate haematologic function was required: a white blood count of ⩾3500 cells *μ*l^−1^, a platelet count of ⩾100 000 *μ*l^−1^, and a haemoglobin level of ⩾10 g dl^−1^. Also, serum bilirubin needed to be below two times the upper limit of normal (ULN), ALT and AST below three times ULN – ALT and AST could be elevated to five times ULN in patients with known metastatic disease in the liver – and creatinine below two times ULN.

Previous chemotherapy was allowed up to second line as long as gemcitabine had not been used. Patients had to be stable on steroids. Patients with seizures who were not stable on antiepileptic medication were excluded. No previous radiotherapy to the brain of any form was allowed. No surgical procedure to the brain and no confusion was allowed (correctable confusion (hypercalcaemia, hyponatremia) with time to correct it and documented adequate mini-mental test scoring was allowed). For female subjects of childbearing potential, adequate contraception during and 3 months post-treatment was required. Patients with haemorrhagic metastases were excluded and patients allergic to contrast material either underwent MRI as baseline study or were excluded.

The protocol was approved by the Hull and East Yorkshire Hospitals Ethics Committee. Written informed consent was obtained from all patients prior to the participation in the study.

CT or MRI scan of the brain was necessary before the beginning of treatment. It was repeated if possible 1 month after the end of the treatment (6 weeks). Patients were withdrawn from the study if they developed sudden neurological deterioration indicating intracranial bleed, allergic reaction to the chemotherapy agent, development of infection allowing continuation of RT but not of chemotherapy and upon patient request.

### Treatment

A conventional radiotherapy fractionation schedule was used (30 Gy in 10 fractions over 12 days). The radiotherapy to the whole brain was planned by simulation and given by parallel opposed right and left lateral portals using six megavoltage X-rays beams. Gemcitabine was given, as an intravenous infusion over 30 min, 2–4 h prior to radiotherapy. If the absolute neutrophil count (ANC) was ⩾750 cells *μ*l^−1^, the platelet count was ⩾100 000 *μ*l^−1^ and the patient had no more than grade 2 nonhaematological toxicity, the full dose was delivered. With an ANC of ⩾500 cells *μ*l^−1^ but < 750 cells *μ*l^−1^ and platelet count of ⩾75 000 *μ*l^−1^ but < 100 000 *μ*l^−1^, 75% of the dose was given. Gemcitabine dose was omitted if platelet count dropped below 75 000 *μ*l^−1^, ANC dropped below 500 cells *μ*l^−1^ or if the patient was experiencing grade 3 or greater nonhaematological toxicity.

The Folstein mini-mental test (Folstein *et al*, 1975) was undertaken at baseline and weekly while on treatment and 4- weekly thereafter until the patient came off study due to progression. It was used as a tool to monitor the cognition of the patients and detect any potential side effects of the treatment to their neurocognitive performance, especially in those who achieved a radiological response or stabilisation of their brain disease.

### Study design

The starting dose of gemcitabine was 25 mg m^−2^ on days 1 and 8. The next level was 25 mg m^−2^ on days 1, 4, 8 and 11 (Table 2).

For dose escalation, three assessable patients had to complete their treatment and the first post-treatment month without a dose limiting toxicity (DLT). Haematological toxicities were defined as DLT if they were grade 4 at WHO scale. All other toxicities were defined as DLT if they were grade ⩾3 at the WHO scale. If no DLT was experienced, then dose escalation could proceed and three patients were enrolled at the next dose level. If two or more patients experience DLT, then the MTD had been defined at this dose level. When one DLT was seen, an additional three assessable patients had to be accrued, and further escalation could occur if no additional DLT were seen. Once the MTD was established, the phase II dose level was defined as the dose level before MTD was reached.

### Response assessment

Response was assessed by repeat CT or MRI scan of the brain 4 weeks after the end of the treatment. We determined response by using the response evaluation criteria in solid tumours group (RECIST) criteria (Therasse *et al*, 2000). Complete response (CR) was defined as disappearance of all target and nontarget tumour lesions and partial response (PR) as a 30% or greater decrease in the sum of the longest diameter of all target lesions together with stabilisation or decrease in size of nontarget lesions. Disease progression (PD) required a ⩾20% increase in the sum of the longest diameter of target lesions, an unequivocal increase in the nontarget lesions or appearance of any new lesions. Stable disease was defined as insufficient tumour shrinkage to qualify for PR and insufficient increase in tumour size to qualify for PD. It has to be noted that the study did not stipulate a further scan 4 weeks later (i.e. 8 weeks post-treatment) to confirm response in responding patients.

## RESULTS

### Patients characteristics

Between February 2001 and July 2003, 25 patients were included in this phase I study. As shown in Table 1, the most common type of cancer was NSCLC, followed by colorectal and breast cancer. In all, 76% of patients had more than one brain metastasis and almost all of them had active malignant disease outside brain (92%). All of them were on oral steroids (dexamethasone). The daily dose ranged from 2 to 16 mg (median dose 12 mg day^−1^) and it remained stable while patients received the study treatment. Two patients were also on oral phenytoin 300 mg day^−1^.

### Dose levels and toxicities observed

Six different doses of gemcitabine were studied. Patients included at the first level of treatment had their gemcitabine doses once per week. All other patients received the chemotherapy drug twice weekly. Overall, 84 doses of gemcitabine were given and the median number per patient was four. Doses up to 37.5 mg m^−2^ were very well tolerated without any signs of significant toxicity. As it can be seen in Table 2 at the dose of 50 mg m^−2^, no DLT has occurred but four out of seven patients needed dose reduction during their treatment. The toxicity that caused dose reduction of the drug was in all cases thrombocytopenia grade 2 during the second week of treatment (third and/or fourth dose of gemcitabine).

At the dose of 62.5 mg m^−2^, one patient developed bone-marrow-related DLT. Neutropenia and thrombocytopenia grade 4 occurred in one of the first three patients treated at this dose. This patient suffered from oesophageal cancer and she was hospitalised but died during the second week of treatment from uncontrolled lower GI bleeding. No further DLTs were seen at this level, but one patient needed a dose reduction due to thrombocytopenia grade 2 and another one developed grade 3 thrombocytopenia after the end of treatment.

Two further patients were treated at the gemcitabine dose of 75 mg m^−2^ and one of them developed grade 4 neutropenia and fatal neutropenic sepsis immediately after the end of treatment. Haematological toxicities are summarised in Table 3. Given the experience at the previous dose level and the further toxicity seen in one patient at dose level 6 (75 mg m^−2^), it was decided that the MTD had been reached at dose level 5 (62.5 mg m^−2^) and the study of further patients at dose level 6 was abandoned. Therefore, dose level 4 (50 mg m^−2^) is the dose to be taken forward to a phase II study. At this dose level, four of seven patients needed dose adjustment of the third and or fourth dose but no greater than grade 2 bone marrow toxicity was noted.

No significant nonhaematological toxicities were observed at any dose level. In particular, no deterioration of the cognitive function of the responding patients was found by using the mini-mental status examination (MMSE). At the beginning of treatment, the average score of patients in the MMSE was 28.5 (range 24–30) with 30 being the maximum score that someone can achieve in the examination. Just after the completion of the treatment the mean score was 28.25 (range 25–30), while at 3 months the median score for six patients who responded to treatment was 29.6 (range 28–30).

### Response rate and survival

Although the aim of this study was not to provide data on these end points, we can report that 12 patients died within the first 2 months from the beginning of treatment and 11 patients were alive more than 5 months later. There were two treatment-related deaths at the two higher dose levels. Of the 13 patients who were alive at 2 months, 11 had a repeated radiological evaluation of their metastatic brain disease. One patient with metastatic rectal cancer showed a complete response (gemcitabine dose 50 mg m^−2^), six patients partial response (four at low doses of gemcitabine, one at 62.5 mg m^−2^ and one at the 75 mg m^−2^ level). In total, three patients had stabilisation of their brain disease.

## DISCUSSION

The results of our phase I study of WBRT with gemcitabine used in a twice weekly schedule as a radiosensitiser in cancer patients with BM is presented.

Gemcitabine was chosen for this study, as it is a potent radiosensitiser *in vitro* and *in vivo*. The retention of the cytotoxic gemcitabine diphosphate (dFdCTP) in cells with a terminal elimination time as long as 72 h is probably a major factor determining this property. The radiosensitisation usually occurs under conditions where cancer cell lines demonstrate a concurrent redistribution in S phase. At this point, depletion of dATP pools (due to ribonucleotide reductase inhibition produced by the drug) leads to misincorporation and misrepair of incorrect bases after radiation (McGinn and Lawrence, 2001; Lawrence *et al*, 2003). It has also been suggested that apoptosis contributes to the radiosensitisation of gemcitabine (Lawrence *et al*, 2001)

These conditions can be reproduced by (i) a long (24 h) exposure to a low concentration of gemcitabine (10 nmol^−l^) or (ii) by a brief 2-h treatment with higher but clinically relevant concentrations (100 nmol^−l^1–3 *μ*mol^−l^) (Lawrence *et al*, 1997). In the second situation, radiosensitisation can be detected 4 h after treatment and can last for 2 days. It is suggested that the active metabolite dFdCTP needs to be present at the time of radiation to potentiate the radiation effects. A twice weekly dosing therefore or a slower rate infusion (10 mg m^−2^ min^−1^) are likely to be preferable as radiosensitisation strategies.

The administration schedule has a profound effect on the gemcitabine dose. This probably again relates to the fact that incorporation of the active metabolite dFdCTP is a saturable process with a prolonged intracellular retention time.

A weekly 30 min infusion can be dosed to up to 2.200 mg m^−2^. Currently, the clinical use of gemcitabine as a weekly single agent is between 1000 and 1250 mg m^−2^. Changing the schedule to twice weekly dosing at the same infusion rate reduces the dose per administration by an order of magnitude. The MTD for twice weekly dosing for 30 min infusions was 65–90 mg m^−2^ (Poplin *et al*, 1992; Lund *et al*, 1994). Interestingly, a review of safety and efficacy data of all European and US studies comparing twice weekly to weekly administration concluded that the actual response rates were similar (Martin *et al*, 1996). However, the twice weekly schedule was not recommended for general use as the reviewers concluded that it caused more ‘flu like’ side-effects (63.3 *vs* 19.8%).

We have demonstrated that gemcitabine on a twice weekly schedule with WBRT can be given at a dose of 50 mg m^−2^. This dose is lower than that reported for single agent gemcitabine (90 mg m^−2^), probably due to the radiotherapy effect despite the fact that the axial skeleton was not irradiated. Similarly to our study, the addition of radiotherapy (to the abdomen for pancreatic cancer) reduced the twice weekly dose further to 40 mg m^−2^ (Blackstock *et al*, 1999). These investigators administered gemcitabine for 5 weeks and the fractionation schedule was 45 Gy in 1.8 Gy daily fractions with a further 5.4 Gy boost. Interestingly even the dose of 60 mg m^−2^ used by Blackstock *et al* (1999) had relatively modest toxicity (only grade 3 bone marrow toxicity noted). In our case, the dose of 62.5 mg m^−2^ had at least one case of severe toxicity. Both in this study and our study, platelet count reduction was the most common toxicity. Although we saw no nonhaematological toxicity, it has to be noted that other studies have shown that depending on the area of irradiation this should be expected. In a study of weekly gemcitabine in head and neck cancer patients (Eisbruch *et al*, 2001), the DLT was severe mucosal and pharyngeal toxicity while in patients with NSCLC DLT was oesophagitis and pulmonary actinic interstitial disease (Trodella *et al*, 2002). We noticed no deterioration in mental function as tested by the Felstein test that could be attributed to treatment despite having patients alive at 15 and 17 months. Nevertheless the majority of the patients had short survival, therefore strong conclusions about potential remote neurological toxicity cannot be drawn especially without a randomised control group for comparison. In contrast to the study by Martin *et al* (1996), we saw no evidence of prominent flu-like symptoms in any of our patients. We have to note, however, that our patients were on high dose (median dose of 12 mg day^−1^) of dexamethasone which may have abrogated this side effect.

To date a number of potential radiosensitising agents such as metallotexaphyrins, synthetic allosteric modifiers of haemoglobin or other chemotherapeutic drugs have been used without a demonstrable improvement in survival of patients with BM (Komarnicky *et al*, 1991; Phillips *et al*, 1995; Mehta *et al*, 2003). Therefore, the investigation of newer promising agents needs to continue.

It would not be safe to reach any conclusions about the efficacy of the addition of gemcitabine as a radiosensitiser to the WBRT. The 44% 5-month survival observed needs to be challenged in a future phase III study comparing the combination with WBRT alone.

We have therefore defined that the MTD of gemcitabine at this schedule in patients with BM is 62.5 m^−2^, and a phase II study with gemcitabine given twice weekly at the preceding dose level of 50 mg m^−2^ as a radiosensitiser to WBRT is already in progress at our centre.

## Figures and Tables

**Table 1:** Patient characteristics (*N*=25)

**Variable**	**Number of patients**	
	***n***	**%**
*Age (years)*		
Median	57	—
Range	41–76	—
		
*Gender*		
Male	15	60
Female	10	40
		
*Performance Status*		
0	6	24
1	18	72
2	1	4
		
*Type of cancer*		
NSCLC^a^	12	48
Colorectal	6	24
Breast	4	16
Other (renal cell and oesophageal)	3	12
		
*Number of brain metastases*		
1	6	24
2 or 3	13	52
4 or more	6	24
		
*Extracranial disease*		
Yes	23	92
No	2	8
		
*Previous chemotherapy*		
Yes	12	48
No	13	52

a NSCLC=non-small-cell lung cancer.

**Table 2:** Dose limiting toxicities (DLTs) and occurrence of toxicities necessitating dose reduction or dose omission by the seven dose levels of gemcitabine tested

**Gemcitabine dose**	**Number of patients treated**	**Number of patients needed reduction or omission of at least one dose**	**Number of patients with a DLT**
25 mg m^−2^ weekly	4	0	0
25 mg m^−2^ twice weekly	3	2	0
37.5 mg m^−2^ twice weekly	3	0	0
50 mg m^−2^ twice weekly	7	4	0
62.5 mg m^−2^ twice weekly	6	2	1
75 mg m^−2^ twice weekly	2	1	1

**Table 3:** Haematological toxicities as occurred among 25 treated patients by the seven dose levels of gemcitabine tested

**Gemcitabine dose**	**Number of patients treated**	**Neutropenia (number of patients)**			**Thrombocytopenia (number of patients)**		
		**Grade 2**	**Grade 3**	**Grade 4**	**Grade 2**	**Grade 3**	**Grade 4**
25 mg m^−2^ weekly	4	1	0	0	1	0	0
25 mg m^−2^ twice weekly	3	0	0	0	0	0	0
37.5 mg m^−2^ twice weekly	3	0	0	0	0	0	0
50 mg m^−2^ twice weekly	7	0	0	0	3	0	0
62.5 mg m^−2^ twice weekly	6	0	0	1	1	1	1
75 mg m^−2^ twice weekly	2	0	0	1	0	1	0
